# Pathogenesis, therapeutic strategies and biomarker development based on “omics” analysis related to microglia in Alzheimer’s disease

**DOI:** 10.1186/s12974-022-02580-1

**Published:** 2022-09-04

**Authors:** Chao Gao, Xin Shen, Yuyan Tan, Shengdi Chen

**Affiliations:** 1 Department of Neurology and Institute of Neurology, Ruijin Hospital, Shanghai Jiao Tong University School of Medicine, Shanghai, 200025 China; 2 Lab for Translational Research of Neurodegenerative Diseases, Shanghai Institute for Advanced Immunochemical Studies (SIAIS), Shanghai Tech University, Shanghai, 201210 China

**Keywords:** Alzheimer’s disease, Microglia, Omics, Pathogenesis, Therapeutic strategy, Biomarker

## Abstract

Alzheimer’s disease (AD) is the most common neurodegenerative disease and the most common cause of dementia. Among various pathophysiological aspects, microglia are considered to play important roles in the pathogenesis of AD. Genome wide association studies (GWAS) showed that the majority of AD risk genes are highly or exclusively expressed in microglia, underscoring the critical roles of microglia in AD pathogenesis. Recently, omics technologies have greatly advanced our knowledge of microglia biology in AD. Omics approaches, including genomics, epigenomics, transcriptomics, proteomics, and metabolomics/lipidomics, present remarkable opportunities to delineate the underlying mechanisms, discover novel diagnostic biomarkers, monitor disease progression, and shape therapeutic strategies for diseases. In this review, we summarized research based on microglial “omics” analysis in AD, especially the recent research advances in the identification of AD-associated microglial subsets. This review reinforces the important role of microglia in AD and advances our understanding of the mechanism of microglia in AD pathogenesis. Moreover, we proposed the value of microglia-based omics in the development of therapeutic strategies and biomarkers for AD.

## Background

Alzheimer’s disease (AD) is the most common neurodegenerative disease and the most common cause of dementia [1]. AD is manifested by memory decline at the early stage and by more severe global cognitive impairments with disease progression. Eventually individuals become bed-bound and require round-the-clock care. However, we still lack effective therapy strategies. The main pathological manifestations of AD include amyloid plaques formed by the deposition of amyloid β (Aβ), neurofibrillary tangles caused by abnormal accumulation of the tau protein, decreased numbers of synapses, and neuronal death in the brain [2].

Previous studies have found that many activated microglia are clustered in close proximity to Aβ plaques in various brain regions (including the cerebral cortex and hippocampus) of AD mice and human postmortem cases [3, 4]. However, these observations do not indicate whether microglia accumulation around the plaques is a cause of the disease or if AD pathology elicits a secondary response by microglia. Recently, genome wide association studies (GWAS) showed that the majority of AD risk genes were highly or exclusively expressed in brain microglia [5]. The R47H variant of triggering receptor expressed on myeloid cells 2 (*TREM2*) increased the risk of developing AD by approximately 2- to 4- fold [6, 7], similar to what has been found in patients with one copy of *APOE* (encoding apolipoprotein E (APOE) ε4). Mutations in other microglial genes, such as *CR1* (encoding complement C3b/C4b receptor 1 (Knops Blood Group)), *CD33*, and *MS4A6A* (encoding membrane spanning 4-domains A6A), were associated with modest risk of AD [5]. Additionally, gene expression network analysis supported the involvement of microglia in the development and progression of AD [5]. Collectively, the above evidence suggests that microglia are critically involved in the pathogenesis of AD, as opposed to being merely a consequence of the response, suggesting microglia as potential therapeutic targets.

Microglia, the resident macrophages of the central nervous system (CNS), perform dynamic surveillance of their microenvironment via their specific receptor repertoire [8]. Microglia maintain homeostasis by phagocytosis to remove cellular debris, dying cells, or misfolded proteins [9]. Microglia could also utilize somatic microglia-neuron junctions to monitor and protect neuron functions [10]. However, microglia are a double-edged sword in AD [11]. On the one hand, microglia phagocytose Aβ and promote Aβ clearance. On the other hand, persistent production of Aβ and its effects on microglia promote Aβ deposition. Aβ-induced pro-inflammatory factors attenuate the ability of microglia to scavenge Aβ [11]. Moreover, Aβ induces activation of the NACHT-, LRR- and pyrin (PYD)-domain-containing protein 3 (NLRP3) inflammasome in microglia, which further promotes the formation and release of apoptosis-associated speck-like protein containing a caspase activation and recruitment domain (CARD) (ASC) specks. ASC specks bind to and cross-seed Aβ after being released from microglia, leading to amyloid seeding and spreading of amyloid pathology [12]. Similarly, the interaction of microglia and tau is also a double-edged sword. Microglia can recognize, engulf, degrade, and clear tau. However, when activated, pro-inflammatory microglia increase tau phosphorylation, and facilitate tau propagation by either trans-synaptic propagation through anatomically connected synapses or via endocytosis and exocytosis [13, 14]. It is argued that microglia play different roles at different stages of disease progression. Microglia might play a protective role to promote misfolded protein clearance in the early stage, and then progress into an irritated state that ultimately becomes deleterious [11]. Additionally, activated microglia mediate synapse loss by engulfment of synapses via a complement-dependent mechanism [15]. However, although studies have emphasized the importance of microglia in AD, our understanding of microglia is still insufficient, which has hindered the development of microglia-targeting therapeutic strategies.

Previously, microglia were classified into two opposite types, the M1 pro-inflammatory phenotype and the M2 anti-inflammatory phenotype, in response to different stimuli in the microenvironment [16–18]. M1 microglia, activated mainly by pathogens and pro-inflammatory factors such as lipopolysaccharide (LPS) and tumor necrosis factor, could release inflammatory cytokines and chemokines, resulting in inflammation and neuronal death. In contrast, M2 microglia are activated by anti-inflammatory factors (e.g., Interleukin (IL)-4, IL-13), leading to the reintroduction of environmental homeostasis and promoting tissue repair [16–18]. However, in recent years, the utility of the M1/M2 classification has been questioned, and the terms M1 and M2 seem to be outdated because they fail to capture the complexity of microglial responses to aging, injury, and disease, and even single stimuli can induce both M1 and M2 responses [19]. It is argued that research has not established microglial polarization [19]. Previously, the ontogeny and functional significance of microglia was not well understood, and the M1/M2 classification was used, which simplified the data interpretation. Lately, omics analysis combined with single-cell technology helped to identify the dynamic changes and heterogeneity of microglia during disease progression. In addition omics technologies have greatly advanced our knowledge of microglia biology in AD. In particular, single-cell RNA sequencing (scRNA-seq) and single-nuclei RNA sequencing (snRNA-seq) analyses have identified special AD-associated microglial subsets in both mouse models and human patient specimens [20, 21]. For example, recent studies have a discovered disease-associated microglia (DAM) subset, which is localized near Aβ plaques, participating in Aβ clearance [21], and a white matter-associated microglia (WAM) subset, which frequently cluster in nodules within the white matter, in which they clear degenerated myelin [22]. Boche et al. reviewed significant recent findings regarding the phenotypic diversity of microglial cells in healthy, aging, and AD brains [23]. The findings implied that the transition from homeostatic microglia to pathological microglia is a dynamic and continuous process involving morphology, motility, metabolism, and proliferation changes [23, 24]. Thus the terminology M1/M2 phenotype should be discarded and microglial states should be defined by their intrinsic and extrinsic determinants, spatiotemporal context, and across multiple omics layers from the genome, transcriptome, and proteome to the metabolome.

**Fig. 1:**
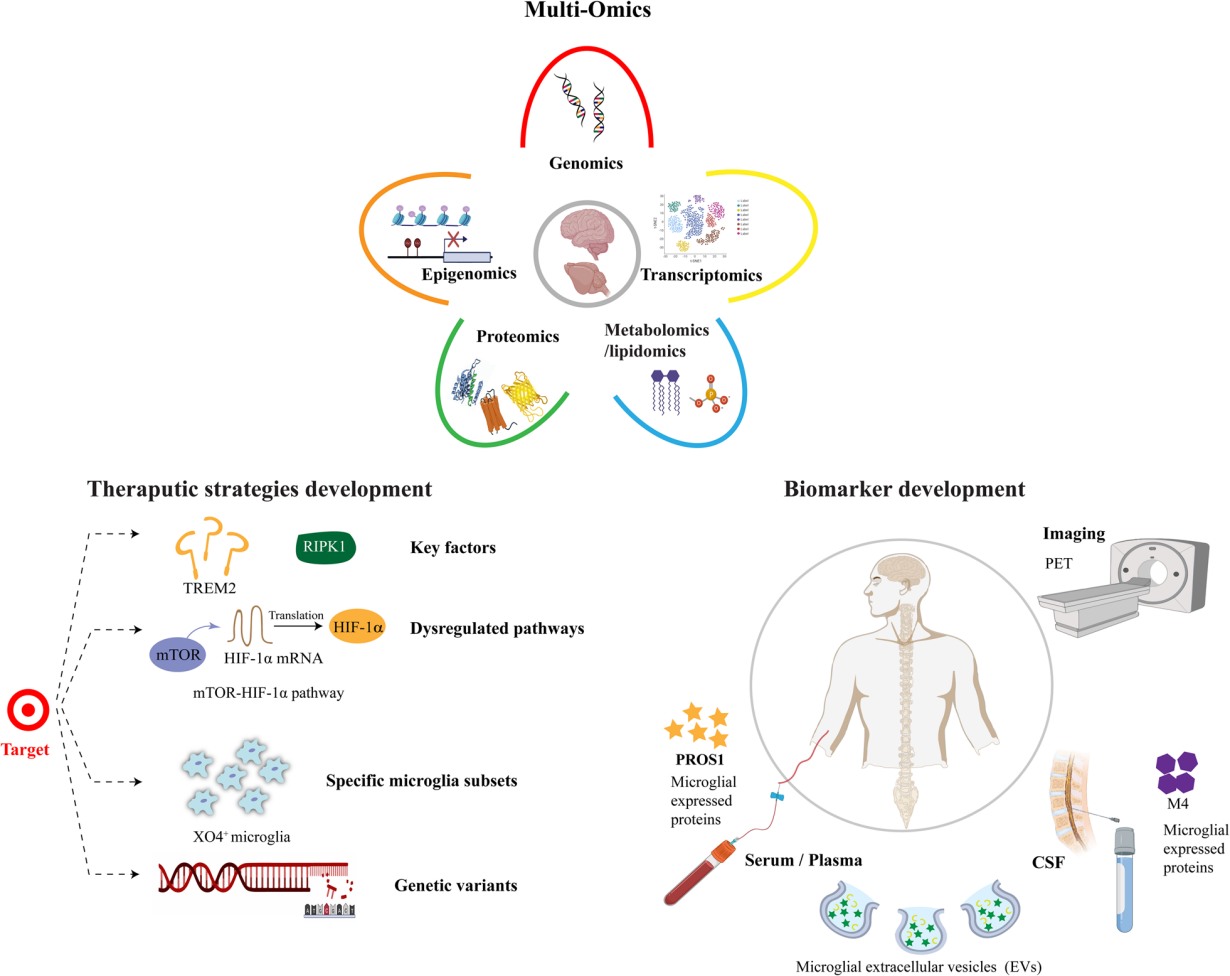
Schematic indicating application of microglial omics in AD. This schematic shows that omics approaches, including genomics, epigenomics, transcriptomics, proteomics, and metabolomics/lipidomics, can be used to delineate the underlying mechanisms of microglia in AD, which is helpful to develop new therapeutic strategies and identify biomarkers to monitor disease progression

In this review, research based on “omics” analysis related to microglia, including genomics, transcriptomics, proteomics, and metabolomics/lipidomics, in AD will be discussed. The findings of these omics studies reinforce the important role of microglia in AD and provide a deeper understanding of the underlying mechanism of microglia in AD, which will help to develop new therapeutic strategies and find biomarkers to monitor disease progression (Fig. 1).

## Clues to the pathogenesis of AD based on “omics” analysis related to microglia

### Genomics

**Fig. 2:**
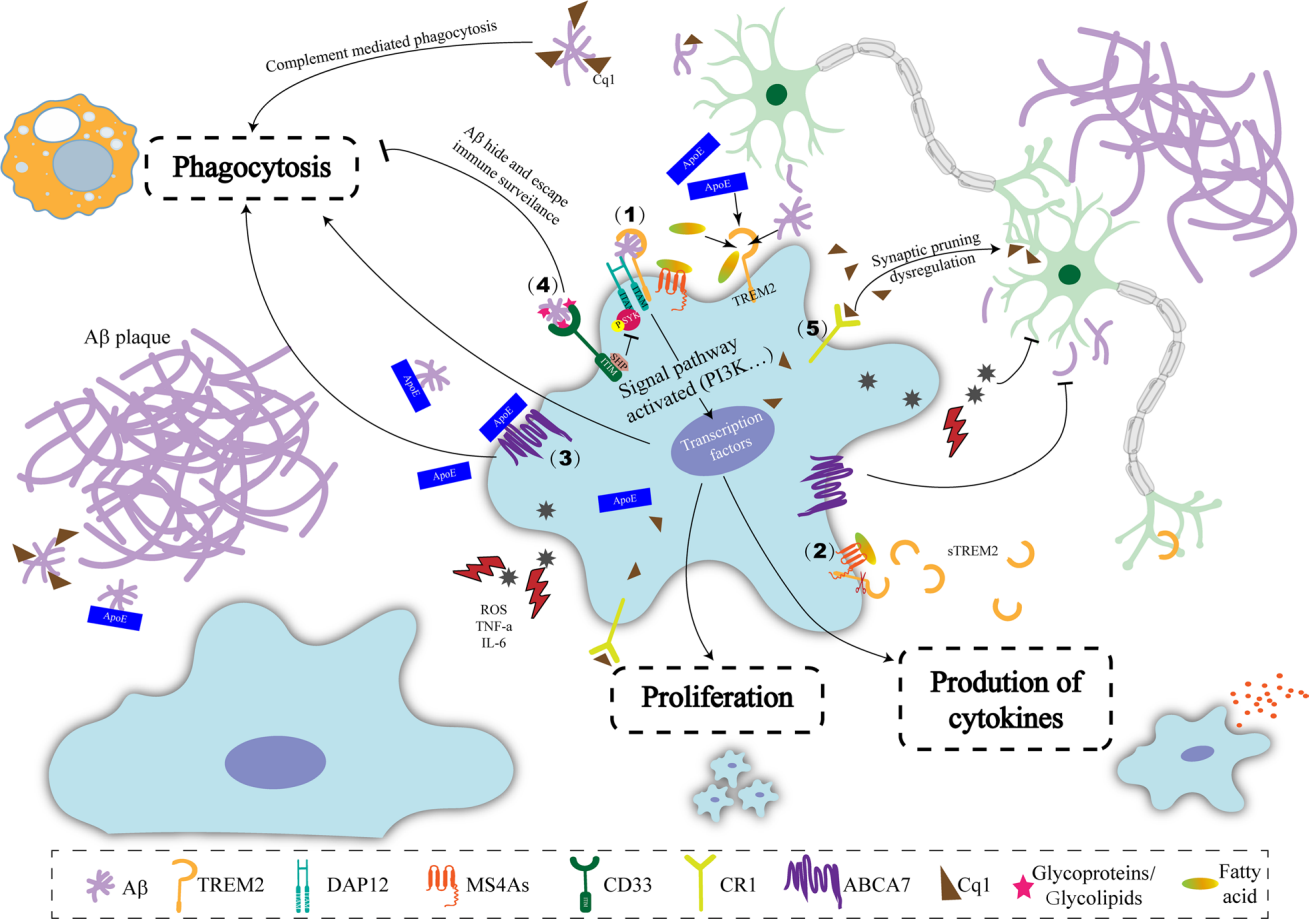
Alzheimer risk genes implicate microglial pathways in AD pathogenesis. (1) TREM2 on microglia binds to extracellular Aβ, ApoE, and lipids, and then interacts with the ITAM in DAP12. This process recruits and phosphorylates SYK. Furthermore, the downstream signaling pathways, including PI3K, are activated and lead to the expression of certain genes, which changes microglial phagocytosis, proliferation, and cell differentiation. (2) MS4As affect the release of sTREM2 and might sense the change of pathological lipids as chemical sensors in combination with TREM2. (3) ABCA7 conserves the function of lipid transport and might transport ApoE in AD. In addition, ABCA7 inhibits Aβ generation and promotes the clearance of Aβ. (4) The ITIM of CD33 combines with SHP, which inhibits the phosphorylation of SYK and the downstream signaling pathways. Furthermore, amyloid plaques decorated by glycoproteins or glycolipids might activate CD33 signaling and then be masked against microglial recognition. (5) CR1 in microglia is involved in the dysregulation of synaptic pruning in AD. However, it also accelerates the clearance of Aβ by complement-mediated phagocytosis. *TREM2* triggering receptor expressed on myeloid cells 2, *Aβ* amyloid β, *ApoE* apolipoprotein E, *ITAM* immunoreceptor tyrosine-based activation motif, *Syk* spleen tyrosine kinase, *ITIM* immunoreceptor tyrosine-based inhibitory motif, *MS4A* membrane-spanning 4-domains subfamily A, *sTREM2* soluble TREM2, *ABCA7* ATP-binding cassette transporter A7, *CR1* complement receptor 1

GWAS have identified more than 40 loci associated with AD [25]. AD risk alleles are specifically enriched in active enhancers of microglia, monocytes, and macrophages [26, 27]. Notably, many of these AD-associated genes (including *TREM2*, *MS4As*, *ABCA7* (encoding ATP binding cassette subfamily A member 7), *CD33*, and *CR1*) are expressed in microglia [5], suggesting that the change of microglial gene expression is involved in the pathogenesis of AD. These AD risk genes that are expressed in microglia have been reviewed in detail previously [5]. Here, we briefly introduced the risk genes supported by relatively more evidence (Fig. 2).

#### TREM2

*TREM2* is expressed highly and exclusively in microglia in the brain [28]. TREM2 suppresses pro-inflammatory cytokine production, facilitates phagocytosis following injury or insult, enhances myeloid cell proliferation, and reduces cell death, which imply multi-faceted roles of TREM2 in maintaining homeostasis in the CNS [29]. Studies of genetic risk for sporadic AD have suggested that coding variants *TREM2* R47H (rs75932628) [6, 7] and R62H (rs143332484) [30] are associated with late-onset AD (LOAD). The studies showed that the rare R47H variant of *TREM2* increased AD risk by approximately 2- to fourfold [6, 7], which was comparable to the effect of the ε4 allele of *APOE* on the risk of sporadic AD. Patients with AD carrying the *TREM2*-R47H or *TREM2*-R62H variant showed defective microglial transcriptional activation and a less evident reactive phenotype [31]. As for the underlying mechanism, microglial TREM2 binds to Aβ, APOE, and various lipids. After binding, TREM2 interacts with the immunoreceptor tyrosine-based activation motif (ITAM) in DNAX-activating protein of 12 kDa (DAP12), initiating the recruitment and phosphorylation of spleen tyrosine kinase (SYK) [32]. Furthermore, downstream signaling pathways including, nuclear factor kappa b (NF-κB) and phosphatidylinositol-4,5-bisphosphate 3-kinase (PI3K), were activated, which led to a change in the phagocytosis, proliferation, and cell differentiation of microglia [33]. In the APP/PS1 transgenic mouse model of AD with *Trem2* knockout or *Trem2* p.T66M mutation, the loss of TREM2 function resulted in impaired microglia phagocytosis ability for Aβ clearance and increased amyloid seeding [34].

#### Membrane-spanning 4-domains subfamily A (*MS4A*)

GWAS reported the association between variants of *MS4A* genes and the risk of AD [35]. A meta-analysis further revealed that the rs610932 G allele and rs670139 T allele increased AD susceptibility both in Caucasian and Asian populations, while rs670139 had no association with AD using genetic models (additive, dominant, and recessive models) in an Asian population [36]. Besides, GWAS found that AD-associated variants rs1582763 (an intergenic variant nearest *MS4A4A*) and rs6591561 (*MS4A4A* p.M159V) were associated with cerebrospinal fluid (CSF) soluble TREM2 (sTREM2) concentrations, which was validated in an independent dataset [37]. An in vitro study found that an antibody targeting MS4A4A significantly reduced sTREM2 concentrations in macrophage cultures [37], which provided a mechanistic explanation of the *MS4A* genetic association with AD risk. In addition to *TREM2* and *MS4A4A*, *MS4A6A* is also exclusively expressed in microglia in the CNS [37]. GWAS identified that rs7232 in *MS4A6A* was associated with CSF sTREM2 levels [38], reinforcing the view that MS4As are involved in the regulation of sTREM2. However, functional studies did not find that MS4A6A could modify sTREM2 [37]. Considering their function, both TREM2 and MS4As sense the lipids in the micro-environment; therefore, it was hypothesized that microglia detected the environmental changes of chemical molecules through MS4As in combination with TREM2 [39].

#### ATP-binding cassette transporter A7 (*ABCA7*)

Several variants in *ABCA7* are associated with AD, as identified in GWAS and meta-analyses [35, 40, 41]. Some studies found that decreased expression of *ABCA7* was associated with increased risk of developing AD [42–45]. Notably, ABCA7 conserves the function of lipid transport activity [46]. The loss of ABCA7 function might contribute to AD pathogenesis by altering proper microglia responses to acute inflammatory challenges [47], disturbing amyloid processing [48], and accelerating Aβ generation [49].

#### CD33

Polymorphisms of *CD33* regulate AD susceptibility and Aβ pathology of LOAD [50–55]. Large scale GWAS identified that rs3865444C was a common allele (> 70%) and was associated with an increased risk of AD [51, 55–57]. This allele produces more full-length CD33 [52–54] and contributes to more severe cognitive deficits in AD [58]. In contrast, the minor A allele of rs3865444 (rs3865444A) decreases the expression of CD33 and increases the proportion of short isoform of CD33, which lacks exon 2, conferring protection against AD [52–54]. In the brain, *CD33* is exclusively expressed by microglia and infiltrating macrophages [52, 53]; therefore, the effects of CD33 on microglia may be critical to the role of CD33-mediated regulation of AD susceptibility. Increased *CD33* expression in microglia impeded the clearance of Aβ by inhibiting microglial phagocytosis [52]. Previous studies indicated several mechanism. Firstly, TREM2 mediates microglial phagocytosis [33] by interacting with the ITAM in DAP12, leading to recruitment and phosphorylation of SYK [32], while CD33 activates the tyrosine-protein phosphatases SHP-1 and SHP-2 [59], which may dephosphorylate SYK; therefore, CD33 potentially antagonizes phagocytic signaling by TREM2. Secondly, full-length CD33 contains an IgV domain that is not present in the short isoform and the IgV domain is crucial for CD33 to suppress amyloid fragment uptake [28]. Thirdly, CD33 might interact with other microglial AD risk factors to influence microglial phagocytosis. For example, TREM2 and CD33 are microglial receptors and TREM2 acts downstream of CD33 in modulating the microglial phenotype in AD [60]. The loss of CD33 attenuated Aβ pathology and improved cognition in 5 × FAD mice (mice expressing human APP and PSEN1 transgenes with five AD-linked mutations) is dependent on TREM2 signaling [60]. Taken together, polymorphisms of *CD33* modulate the expression level of CD33 and the ratio of full-length CD33 to short isoform CD33, which in turn alters the phagocytic capacity of microglia, ultimately exerting deleterious or protective effects in AD.

#### Complement receptor 1 (CR1)

Multiple GWAS implied that several single nucleotide polymorphisms (SNPs) within or around *CR1* were associated with LOAD [61–63]. Some studies showed that *CR1* polymorphisms increased the risk of LOAD [64–67]. In the periphery, *CR1* is expressed on immune-related cells in the blood and binds to fragments of complements to activate the complement cascade. Previous studies reported that CR1 could also be found in microglia in the human brain [68]. CR1 promotes the clearance of Aβ through microglial phagocytosis mediated by complements [69–71]. Additionally, CR1 might play a role in excess synaptic pruning in AD. Evidence showed that direct opsonization and elimination of synapses by C1q is likely mediated through CR1 [72] and C1q-mediated synaptic pruning is inappropriately activated, leading to excess synapse loss in AD [73]. Besides, *CR1* is widely expressed in many cell types both in the periphery and in the brain; therefore, it is possible that the role of CR1 in LOAD could be mediated by other pathways besides microglia.

#### Bridging Integrator 1 (*BIN1*)

GWAS has identified *BIN1* as a significant genetic risk factor locus for LOAD [40, 74]. *Bin1* was highly expressed in microglia of wild-type mice and mouse models of AD pathology, as measured by quantitative mass spectrometry [75–77]. The *BIN1* enhancer region contains the AD-risk variant rs6733839, which has the second highest AD-risk score after APOE. CRISPR/Cas9-mediated deletion of the *BIN1* enhancer ablated *BIN1* expression in human pluripotent stem cell (PSC)-derived microglia, but not in human PSC-derived neurons and astrocytes [27]. This finding that the most significant GWAS risk allele associated with *BIN1* resides in a microglia-specific enhancer indicates that abnormal expression of *BIN1* in microglia is probably associated with AD pathogenesis. Mechanistically, BIN1 is a key regulator of microglia activation and the proinflammatory response. In vitro and in vivo studies involving silencing *Bin1* expression in primary mouse microglia found that BIN1 regulated the activation of proinflammatory responses upstream of *Apoe, Trem2, and Tyrobp* (encoding TYRO protein tyrosine kinase binding protein), and upstream of *Pu.1* (encoding Spi-1 proto-oncogene) and *Irf1* (encoding interferon regulatory factor 1), which mediated the transition to DAM [78]. Conditional knockout of microglial *Bin1* mitigated LPS‑mediated proinflammatory activation and the DAM gene expression profile in mice. BIN1 was also found to regulate inflammation-induced expression of *Ifitm3* [78], an interferon-response gene encoding interferon induced transmembrane protein 3, which plays a role in microglial inflammatory responses to AD pathology [79]. LPS-induced upregulation of *Ifitm3* was significantly impaired in the absence of *Bin1* expression [78]. Additionally, BIN1 overexpression facilitated the release of tau via extracellular vesicles in vitro and aggravated tau pathology in vivo. *Bin1* knockout in the microglia of male mice significantly reduced the expression of heat-shock proteins, which were previously implicated in tau proteostasis, suggesting that microglial BIN1 might also affect tau clearance [80]. Taken together, BIN1 is involved in AD pathogenesis by regulating microglia activation and proinflammatory response, altering tau clearance, and promoting release of tau-enriched extracellular vesicles by microglia.

#### PU.1

*SPI1* encodes PU.1, a transcription factor critical for myeloid cell development in the brain and periphery [81, 82]. In the brain, PU.1 is specifically expressed in microglia and recent evidence from GWAS suggests that the minor allele of rs1057233 (G), which lowers the expression of PU.1, showed association with delayed AD onset [83]. Experimentally altered PU.1 levels affected the expression of many AD-associated microglial genes involved in the innate and adaptive immune systems [83, 84], indicating that PU.1 is a key hub protein in regulating the microglial immune response in AD pathogenesis. PU.1 is required for microglial activation in response to neurodegeneration. Mechanistically, PU.1 and insulin response factor 8 (IRF8) were upregulated during microglial activation by directly targeting each other's gene transcription in a positive feedback loop. PU.1 cooperates with IRF8 to bind to the composite IRF-ETS motifs that are specifically enriched on microglial activation-related genes [85]. Hence, PU.1 is implicated in the central transcriptional mechanism of microglial activation in response to neurodegenerative conditions.

#### Phospholipase C gamma 2 (*PLCG2*)

Recent identification of the rare LOAD-protective variant (rs72824905, p.P522R) has established *PLCG2* as a new AD-linked gene [86]. *PLCG2*, encodes thephospholipase C gamma 2 (PLCγ2) enzyme, which catalyzes the conversion of membrane phospholipid PIP2 (1-phosphatidyl-1D-myo-inositol 4,5-bisphosphate) into IP3 (myo-inositol 1,4,5-trisphosphate) and DAG (diacyl-glycerol). In turn, IP3 and DAG regulate microglial phagocytosis, cytokine production, and survival [87]. PLCG2 is upregulated in the brains of patients with LOAD and in 5 × FAD mice [88]. The expression level of *PLCG2* correlated significantly and positively with amyloid plaque density [88]. PLCγ2 is expressed specifically in microglia within the CNS, acting as downstream effector of Toll-like receptors to mediate inflammatory responses [89]. PLCγ2 also acts downstream of TREM2-DAP12 signaling via interaction with SYK, thus mediating cell survival, phagocytosis, processing of neuronal debris, and lipid metabolism [89]. A PLCγ2-P522R knock-in mouse model was established to assess the role of the protective variant in immune cells, which showed that the PLCγ2-P522R variant promoted microglial immune functions [90]. Besides, the PLCγ2-P522R variant increases the capacity of human microglia to present antigens and promoted the recruitment of CD8 + T cells to the brain in AD mouse models [91]; however, the role of the PLCγ2-P522R variant in the crosstalk between microglia and T cells in AD requires further study.

Notably, AD-risk variant SNPs do not always affect the function of their closest genes. In fact, some enhancer-located SNPs influence the expression of surrounding or distant genes by contacting gene promoters and regulating specific transcription factor activities via chromatin higher-order structures [92]. Moreover, in AD, some of these three-dimensional interaction-dependent extended gene regulation networks between enhancers and promotors are microglia specific [27]. For instance, the *SLC24A4* locus (encoding solute carrier family 24 member 4), includes AD-risk variants that were linked to the proximal active promoters of *ATXN3*, *TRIP11*, and *CPSF2*, but not to *SLC24A4* via chromatin loops. Another example is that AD-risk variant rs6733839 is located in the *BIN1* enhancer, which interacts with the *BIN1* promoter by chromatin conformation changes. Deletion of the *BIN1* enhancer harboring AD-risk variant rs6733839 ablated *BIN1* expression in microglia but not in neurons or astrocytes [27].

Overall, many AD risk genes are highly expressed in microglia and participate in multiple levels of microglia functions, such as microglia activation, toxic protein clearance by microglia, synaptic pruning of microglia, and dynamic surveillance of the microenvironment. These risk genes might have tight functional connections and work together to regulate microglia responses. Moreover, those AD risk genes that are expressed in microglia could also have impact on other AD risk genes that are not expressed in microglial cells, for example, a mutated *SORL1* allele, encoding the sortilin related receptor 1 R744X mutant, induced *TREM2* expression to enhance *APOE* expression [93]. Gene coexpression networks that are enriched with genetic signals and are involved in the immune response for AD have been identified. Some investigators have used gene networks and computational drug repositioning analyses to screen candidate drugs for AD therapy [94]. Moreover, gene coexpression networks can link biological pathways to specific pathological changes, e.g., some AD related risk genes determine the microglial response to Aβ [95].

### Epigenomics

Epigenetic studies investigate the mechanisms that modify the expression levels of genes at the transcriptional level. There is growing evidence for the prominent role of DNA methylation in AD [96]. A meta-analysis including 1030 prefrontal cortex samples from four independent cohorts found that 3751 CpGs and 119 differentially methylated regions (DMRs) were significantly associated with AD Braak stage [97]. The most significant DMR was in the promoter region of the MEF2-activating motif and SAP domain-containing transcriptional regulator (*MAMSTR*) gene [97]. MAMSTR is a transcriptional coactivator that stimulates Myocyte enhancer factor-2C (MEF2C). TNF-injected mice lacking *Mef2C* in their microglia showed an exaggerated microglial response and had an adverse effect on mice behavior [98], suggesting that MEF2C might function as a main constraint on the microglial over-response to immune stimuli in the context of AD. Moreover, MEF2C cooperates with another transcription factor, PU.1, to regulate the promotor activity of specific genes such as that encoding the immunoglobulin J chain [99]. In the brain, PU.1 is specifically expressed in microglia and as a transcriptional factor, regulates AD-associated genes in primary human microglia, such as *SPI1*, *TYROBP*, and *TREM2* [84]. Therefore, abnormal methylation regulation might affect the expression of *MAMSTR* and its functional role in immune regulation. Dysregulated *MAMSTR* expression might also interfere with the binding of PU.1 to its target genes and the gene expression network, promoting the development of AD. These hypotheses need to be verified by further experiments. Although *APOE* ε4 increases the risk for AD, not all ε4 carriers develop AD, indicating that there are some factors that might attenuate the risk of ε4 on AD. Ma et al. conducted a meta-analysis to explore the epigenome of the human brain for CpG dinucleotides that attenuate the impact of *APOE* ε4 on the risk of AD in the general population harboring the *APOE* ε4 haplotype [100]. DNA methylation of four CpG dinucleotides on different chromosomes (cg08706567 at *MPL* (encoding myeloproliferative leukemia protein), cg26884773 at *TOMM20* encoding translocase of outer mitochondrial membrane 20), cg12307200 between *LPP* (lipoma preferred partner) and *TPRG1* (tumor protein p63 regulated 1), and cg05157625 at *RIN3* (Ras and Rab interactor 3)) were identified to be associated with AD susceptibility in *APOE* ε4 + individuals. In downstream analyses, they found that the identified CpG dinucleotides attenuated the risk of ε4 on AD probably through reduced microglial activation in the brain [100]. Overall, epigenetic changes, including DNA methylation or histone modifications, are important modifiers of gene expression, and are involved in the microglial cell phenotype regulation [101]. In AD brains, microglia possess disease-specific epigenomes and associated transcriptomes, which have impacts on microglia biology. Investigation of the epigenetic machineries could provide interesting targets for the treatment of AD.

### Transcriptomics

#### Microglial subsets reported in AD mouse models

Recently, the development of scRNA-seq and snRNA-seq technologies paved the way for identifying microglia subsets, and the emerging microglia subsets greatly advanced our knowledge of microglia responses in AD. For example, Keren-Shaul et al. first performed scRNA-seq in the 5 × FAD AD transgenic mouse model and identified a subgroup of microglia in AD, termed DAM [21]. Microglia displayed a transition from homeostatic condition to DAM with disease progression in the 5 × FAD mice. The authors identified that the transition was regulated in a two-step process. The first step, which is TREM2-independent, involves reduction in the expression of homeostatic microglia checkpoint genes such as *Cx3cr1* and *P2ry12/P2ry13*, and activation of a set of genes including *Tyrobp, Apoe,* and *B2m*. The second step, which is a TREM2-dependent pathway, involves upregulation of phagocytic and lipid metabolism genes such as *Cst7* and *Lpl*, corresponding to the need for plaque clearance in AD [21]. scRNA-seq analysis in *Trem2*^+*/*+^ versus *Trem2*^*−/−*^ AD mice demonstrated that the microglia in the intermediate state, but not the final state, were much more abundant in the *Trem2* knockout AD mice, indicating TREM2 plays a crucial role in the transition of microglia from the intermediate to the final state [21]. DAM were localized near Aβ plaques, which has also been validated in AD post-mortem brain samples [21]. DAM participated in the clearance of Aβ, suggesting their protective role in AD [21]. Notably, the above DAM signature was quite distinct from the previously categorized M1/M2 polarization phenotype [102], thus it was suggested that scRNA-seq has started a new chapter in our understanding of microglia biology. The DAM-like subset was also found in mouse models of other neurodegenerative diseases, such as amyotrophic lateral sclerosis (ALS) and multiple sclerosis, which was termed the microglial neurodegenerative phenotype, MGnD [103]. The MGnD phenotype was characterized by the loss of homeostatic genes and upregulation of inflammatory molecules, of which *Apoe* was one of the most upregulated genes. Induced by phagocytosis of apoptotic neurons, homeostatic microglia convert into the MGnD signature through the TREM2-APOE pathway. Genetically ablated *Trem2* in AD and ALS mice suppressed APOE signaling and restored the homeostatic signature of microglia, indicated that targeting the TREM2-APOE pathway might provide a therapeutic strategy for neurodegenerative disorders [103]. Besides, Mathys et al. analyzed the dynamic changes of microglia during disease progression in the CK-p25 mouse model [104], which exhibited elevated Aβ levels, progressive neuronal death, reduced synaptic plasticity, and cognitive impairment [105]. In the early period of the disease, the microglia showed increased proliferation, while at the later stage of neurodegeneration, two distinct reactive microglia, the IFN-I subset, which expressed IFN-I-induced genes, and the MHC-II subset with strong expression of *MHC-II* genes, were present [104]. Whether the induction into these two distinct reactive microglia phenotypes in the CK-p25 model is protective, neutral, or deleterious remains to be determined. Recently, white matter-associated microglia (WAM) were discovered in a mouse model of AD [22]. WAM were characterized by downregulation of homeostatic genes and activation of phagocytic and lipid-metabolism-related genes. A WAM signature was also identified in previous scRNA-seq datasets [106, 107], indicating its robustness and reproducibility. WAM formation depends on age, and TREM2 and APOE-mediated signals. WAM function to clear degenerated myelin [22].

#### Microglial subsets reported in patients with AD

Microglia subsets have also been discovered in patients with AD. A recent study performed snRNA-seq of the occipital cortex which contained Aβ pathology, with no or low-level tau-pathology, and the occipitotemporal cortex, which contained both Aβ pathology and tau pathology, from patients with AD and controls [20]. This identified three populations: homeostatic microglia, AD1-microglia, and AD2-microglia. AD1-microglia correlated strongly with the tissue Aβ load and were localized to Aβ plaques. Gene ontology analysis indicated that AD1-microglia were associated with ‘cell migration’, ‘phagocytosis’, and ‘lipid localization’, which are similar to the DAM signature in AD mouse models. AD2-microglia possibly have neurotrophic functions; however, that study did not examine the functional role of AD1- or AD2- microglia [20]. Besides, Nguyen et al. characterized four microglia subpopulations: dystrophic microglia, amyloid-responsive microglia (ARM), homeostatic microglia, and motile microglia, in which the ARM subset depended on TREM2 and APOE signaling, because the ARM subsets were lost in cases with APOE and TREM2 risk variants [108]. Interestingly, discrepancies have been observed between human and mouse microglia signatures. Zhou et al. found that in AD, human microglia presented partial DAM signatures such as upregulation of *TREM2*, *APOE*, *CD68*, and *HLA-DRA*, whereas other DAM-related genes were undetected or downregulated [31]. A microglia subset identified in patients with AD showing upregulation of previously identified homeostatic genes in mice (*TMEM119*, *P2RY12*, and *CX3CR1*), along with higher expression of the transcription factor interferon regulatory factor 8 (*IRF8*) was remarkably similar to the IRF8-driven reactive microglia phenotype in the mouse peripheral nerve injury model. In vitro studies also found that IRF8 drove the expression of microglial markers linked to AD [31], which suggested that IRF8 might trigger the microglia signature transition in the context of AD. The reasons for the discrepancies between human and mouse microglia profiles have been recently discussed elsewhere, including both biological and technical reasons [102, 109]. Summaries of microglial subsets reported in AD mouse models and patients with AD are listed in Tables 1 and 2.

**Table 1:** Summary of microglial subsets reported in AD mouse models

Disease model	Methods	Subsets	Signature	Conversion	Function	Publication
5 × FAD	scRNA-seq	DAM	↓: P2ry12/P2ry13, Cx3cr1, Tmem119 ↑: ApoE, Ctsd, Lpl, Tyrobp and Trem2	Two-Step Activation Mechanism: First step, Trem2-independent; Second step, Trem2-dependent	Participating in the clearance of amyloid β	Keren-Shaul et al. [21]
4-month-APP/PS1	Single-cell mass cytometry with fluorescence cytometry	Confirm the DAM	↑: CD11c and CD14			Mrdjen et al. [184]
SOD1G93A APP/PS1, EAE mice	RNA-seq	MGnD	↓: Homeostatic genes ↑: Inflammatory molecules, such as ApoE	Trem2-APOE pathway		Krasemann et al. [103]
CK-p25	scRNA-seq	Proliferating microglia (early stage)				Mathys et al. [104]
		MHC-II (later stage)	↑: Class II components genes			
		IFN-I (later stage)	↑: Type I interferon response gene			
Trem2-/-, 18–20 months old, 6-month-old 5 × FAD	scRNA-seq	WAMs	↓: Homeostatic genes and checkpoint genes ↑: DAM-associated genes	In aged mice: age and Trem2 dependent; In AD model: age and Trem2, ApoE dependent	Clearing degenerated myelin	Safaiyan et al. [22]
PS2APP, 5 × FAD, APPswe/PS1De9 and two tau models: P301S/L	RNA-seq	Confirm the DAM and IFN-I				Friedman et al. [183]
		Proliferating microglia	Expressing proliferation module ↑:slightly: mitosis genes			

*AD* Alzheimer’s disease, *scRNA-seq* single-cell RNA sequencing, *DAM* disease-associated microglia, *MGnD* microglial neurodegenerative phenotype, *IFN-I* type I interferons, *WAMs* white matter-associated microglia, *TREM2* triggering receptor expressed on myeloid cells 2

**Table 2:** Summary of microglial subsets reported in AD patients

Subjects	Methods	Subsets	Signature	Conversion	Function	Publication
482,472 nuclei from 18 non-demented control brains and AD brains	snRNA-seq	Homeostatic microglia	Expressing P2RY12 and CX3CR1			Gerrits et al. [20]
		AD-1 microglia	↑: Phagocytic associated gene, DAM-like genes	Response to amyloid-β in the extracellular space		
		AD-2 microglia	↑: GRID2	Response to p-tau bearing (dying) neurons		
131,239 nuclei from 48 cases	snRNA-seq	Dystrophic microglia	↑: Pro-inflammatory related genes ↑: FTL and FTH1			Nguyen et al. [108]
		Amyloid-responsive microglia (ARM)	↑: Pro-inflammatory related genes ↑: CD163, BIN1, MS4A6A, and CELF1	ARM subsets depend on APOE and TREM2 signaling		
		Homeostatic microglia	Expressing CX3CR1			
		Motile microglia	↑: Genes associated with cell motility, actin remodeling, and extracellular matrix remodeling			
66,311 nuclei from 11 AD with the TREM2-CV, 10 with TREM2-R62H and 11 controls	snRNA-seq	IRF8-driven reactive microglia	↑: TREM2, APOE, CD68, and HLA-DRA (Partial DAM) ↑: IRF8, SORL1, A2M and CHI3L1 ↑: Homeostatic gene TMEM119, P2RY12, and CX3CR1 ↓:SPP1	IRF8 is likely a major driver of this signature		Zhou et al. [31]

*AD* Alzheimer’s disease, *snRNA-seq* single-nuclei RNA sequencing, *DAM* disease-associated microglia, *ARM* amyloid-responsive microglia, *IRF8* transcription factor interferon regulatory factor 8, *TREM2* triggering receptor expressed on myeloid cells 2

Currently, except for microglia subsets such as DAM and WAM, most microglia subsets were only defined by transcriptional profiling; however, their cellular functions that positively or negatively contribute to AD pathogenesis remain unclear. Understanding the functional roles of these subsets and identifying regulatory factors of specific microglia subsets will benefit AD therapy.

### Proteomics

Proteomics is the analysis of the entire protein complement of a cell, tissue, or organism in the context of a specific, defined set of conditions, resulting in an information-rich landscape of expressed proteins and their modulations. Rayaprolu et al. used proteomics to analyze the fluorescence-activated cell sorting (FACS)-isolated microglia proteome and the magnetic-activated cell sorting (MACS)-enriched microglia proteome from the brains of adult wild-type (WT) mice. A total of 203 consensus microglial proteins in both datasets were identified, in which moesin (Msn) was highly expressed [110]. Further studies showed the Msn, a member of the ERM ((ezrin, radixin, and moesin) family that connects the actin cytoskeleton to the plasma membrane [111], was highly expressed in microglia that surrounded Aβ plaques in both AD mouse models and human AD brains. Msn probably plays a protective role in AD by mediating Aβ phagocytosis and decreased neuroinflammation. In control, asymptomatic AD cases (AsymAD, which had postmortem AD pathology but without dementia), and AD cases, the protein level of Msn in the precuneus region displayed a strong positive correlation with Aβ plaque and neurofibrillary tau tangle and a negative correlation with cognitive function [110]. Recently, in a large proteomic study of the post-mortem brains of over 400 patients with AD, a consensus network of protein co-expression modules demonstrated Msn as a hub protein [112], indicating a key role of Msn in AD pathogenesis. Similarly, Cotl1, an actin binding protein, was first identified by proteomics as a novel microglia-specific marker [113]. Cotl1was highly expressed in purified CD11b^+^ acutely isolated microglia from an AD mouse model and was further validated in morphologically-activated microglia derived from the frontal cortex of patients with AD. Cotl1 also showed a strong positive correlation with neurofibrillary tangle pathology [113]. Notably, although the protein levels of Cotl1 and Msn correlated positively with microglia activation, their levels were only increased in patients with AD, but not in those with ALS or Parkinson’s disease (PD) [110], despite microglia activation being regarded as a common immune response in neurodegenerative diseases [11]. These findings indicated a unique microglia activation signature in AD.

Morshed et al. identified signaling pathways that were dysregulated in various mouse models of AD using quantitative phosphoproteomics and found that Siglec-F was upregulated as a shared response in a subset of reactive microglia [114]. The levels of the human paralog, Siglec-8, were also increased in aged human microglia and in the microglia of patients with LOAD [114]. Previous studies showed Siglec-F is an eosinophil surface receptor, and Siglec-8 is expressed by human eosinophils. Genetic knockout of *Siglecf* lead to increased inflammation in asthma [115], indicating that Siglec-F mediated an immune response. An in vitro study showed that both Siglec-F and Siglec-8 were upregulated following microglial activation, and *Siglecf* overexpression activated an endocytic and pyroptotic inflammatory response in BV-2 cells, a mouse microglial cell line [114]. The functions and mechanisms of Siglec-F and Siglec-8 are unclear; therefore, whether their upregulation on microglia during aging and AD are secondary to microglial activation, or whether they mediate microglial inflammatory pathways, requires further investigation.

The responses of microglia to AD pathology comprise dynamic changes during various stage of disease progression. A recent study performed an in-depth and time-resolved proteomic analysis and identified a large panel of microglial Aβ response proteins whose levels changed in parallel with microglial alterations during the early, middle, and advanced stages of Aβ deposition in two mouse models of Aβ pathology [116]. The dynamic changes characterized in the proteomic profiles of microglia of AD provide a valuable resource for new therapeutic strategies and the development of biomarkers to monitor AD progression.

### Metabolomics/lipidomics

Metabolomics is defined as the comprehensive analysis of metabolites in a biological specimen. Metabolites are the substrates and products of metabolism that drive essential cellular functions. Thus, metabolomics is an emerging but powerful tool to provide insight into the mechanisms that underlie various physiological conditions and diseases. TREM2 has been identified as a risk factor for LOAD [6, 7]. TREM2 was strongly implicated in microglial phagocytosis to remove dead neurons, damaged myelin, and Aβ plaques [117, 118]. However, why the loss of TREM2 function resulted in impaired microglia phagocytosis ability for Aβ clearance [34] is not known. Using electron and confocal microscopy to analyze microglia, it has been revealed that microglia in patients with AD patients carrying *TREM2* risk variants and a *Trem2*-deficient AD mouse model have abundant autophagic vesicles [119]. Metabolomics combined with RNA sequencing (RNA-seq) linked those abundant autophagosomes to defective mTOR signaling, which affected biosynthetic pathways and ATP levels. Dietary supplementation with cyclocreatine, which can generate a supply of ATP for energy demands in *Trem2*-deficient mice with Aβ pathology, rescued microglial clustering around plaques, and ameliorated plaque adjacent neuronal dystrophy [119]. Thus, metabolomics facilitates the uncovering of the mechanism of TREM2's critical role in sustaining cellular energetic and biosynthetic metabolism of microglia to enable their responses during AD.

Lipidomics is considered a subfield of metabolomics. TREM2 can bind to lipids and promote the phagocytic uptake of lipid-rich myelin [22, 120]; however, its role in microglial lipid metabolism is unknown. Combining chronic demyelination paradigms and cell sorting with RNA-seq and lipidomics, Nugent et al. found that *TREM2*-deficient microglia could phagocytose myelin debris, but fail to clear myelin cholesterol, resulting in cholesteryl ester accumulation [120]. This finding was helpful to reveal the mechanism by which TREM2 regulates cholesterol transport and metabolism in microglia under conditions of chronic myelin phagocytic activity. Thus, by analyzing the substrates and products of metabolic processes, metabolomics might reveal dysregulated metabolic pathways of microglia in the pathogenesis of AD and promote investigations to develop novel approaches that modulate microglial metabolism to slow down disease progression.

### iPSCs as a powerful tool for “omics” study in AD

Human induced pluripotent stem cells (hiPSCs) have been used to investigate the molecular mechanisms underlying AD. iPSC-derived microglia can phagocytose Aβ or tau, two hallmark AD pathologies [121]. Moreover, once exposed to fibrillar Aβ, iPSC-derived microglia increased the expression of several AD-GWAS related genes, such as *TREM2* and *APOE*, which were induced in disease-associated microglia in AD brains and were implicated in Aβ clearance or degradation [121]. Recent studies also analyzed the effect of APOE4 on gene expression in iPSC-derived microglia and the ability of microglia to phagocytose Aβ. It was found that, compared with *APOE3*, *APOE4* variants are more likely to induce microglial inflammatory gene activation and reduce microglial Aβ uptake, both of which are associated with AD development [122]. Similarly, iPSC-derived microglia harboring microglia-associated AD-risk gene *TREM2* missense mutations showed marked impairment of the phagocytosis of apoptotic bodies [123]. This evidence validated the feasibility of AD modeling using the iPSC microglial models because they can capture the relevant biology of AD, such as the Aβ-induced DAM phenotype and impaired phagocytosis with AD-related variants. In the future, iPSC-derived microglia could be used for high-throughput screening of drugs that enhance the phagocytosis of Aβ. More importantly, combined with omics analysis, it is beneficial to clarify the mechanisms of GWAS-identified AD-related variants in the occurrence and development of AD (see the example in Sect. 2.7).

### Advantages and limitations of integrative multi-omics

Multi-omic analyses at the bulk microglia level provides a comprehensive understanding of cellular processes through the integration of different types of molecular data from genomics, epigenomics, transcriptomics, proteomics, and metabolomics/lipidomics. For example, GWAS have identified many genetic variants associated with AD; however their functional roles are rarely identified. Liu et al. integrated multi-omic analysis including ATAC-seq (assay for transposase-accessible chromatin with high-throughput sequencing), ChIPseq (chromatin immunoprecipitation sequencing), RNA-seq, and proteomics combined with an individual omics integrator network to study the regulatory role of AD variants in *CD33*, *INPP5D*, *SORL1*, and *TREM2* loci in isogenic human embryonic stem cell (ESC)-derived microglia-like cell lines and identified upregulation of APOE as a convergent pathogenic node [93]. Recent advances in single-cell isolation and barcoding technologies have enabled mRNA, protein, lipid, and metabolite profiles to be measured at a single-cell resolution, which, in combination with multi-omic analysis, promotes the comprehensive elucidation of complex biological processes of microglia in the content of AD. For example, Cohn et al. identified that the loss of the homeostatic microglia signature in late AD stages was accompanied by endolysosomal impairment and the release of neuronal and myelin debris using an integrated analysis of proteins, lipids, and miRNAs of isolated microglial extracellular vesicles from cryopreserved human brain tissue [124]. Therefore, single-cell multi-omic analysis can provide more comprehensive insights into microglia-specific gene regulation than bulk microglial omics or single-cell mono-omic analysis. Integrative analysis of single-cell genome and transcriptome data can demonstrate the link between genomic variants and the transcription of target genes. Integrative analysis of the epigenome and transcriptome can reveal the regulatory role of epigenetic modifications on the expression of target genes. Integrative analysis of single cell RNA-seq (scRNA-seq) and mass cytometry (cytometry by time-of-flight [CyTOF]) provides insights into cell signaling dynamics in targeted cells. Emerging microglia subsets in both AD mouse models and patients with ADs have been identified; however, the driving factors responsible for the emergence of a specific subset of microglia remain unclear. In the future, using computational methods to analyze multi-omic data will help to more accurately predict the transcription factors involved in the regulation of specific subgroups, which will be beneficial to the development of therapeutic targets.

Notably, some limitations of single-cell techniques or multi-omic analyses need to be considered when interpreting the results and drawing conclusions [125]. Firstly, there is a measurement bias caused by technical limitations. Even the most sensitive scRNA-seq protocols detect only around 10% of the transcriptome [126, 127]. Moreover, the number of proteins that can be detected is limited because of the insufficient detection sensitivity of current single-cell multi-omic techniques. Cell fixation or post-mortem brain tissue further reduce the yield or quality of RNAs and proteins for omic-analyses, tending to lead to measurement bias. Secondly, measurement bias can also be introduced when processing samples. For instance, tissue dissociation, including enzymatic digestion and mechanical dissociation, might sometimes cause microglia activation, presented as an immediate early gene signature [128, 129]. Bisulfite treatment might cause DNA damage that can affect the accuracy of the DNA methylome measurement. Thirdly, human brain tissue samples are generally frozen or paraffin-embedded, and the freezing process disturbs the cytoplasmic membrane, while the nuclear membrane remains intact. This will result in a lack of information for cytosolic mRNAs, which could lead to misleading conclusions, although the analysis of genomic DNA and nuclear mRNA after the isolation of single-cell nuclei is still possible [125]. Besides, computational methods for the integrative analysis of single-cell multi-omic data is at the very early stage. In the future, advances in experimental technologies and data analysis methods are needed to ensure more accurate identification of biological alterations in disease pathogenesis. These molecular mechanisms can be further used to develop new diagnostic markers and therapeutic targets.

## Therapeutic strategies based on microglial “omics” analysis in AD

### Targeting key factors and pathways to improve microglia function

Omics research provides a powerful approach to screen the key factors and pathways that are changed or dysregulated in microglia in the brains of AD mouse models and patients with AD. Moreover, interventions for these targets could probably treat the disease effectively or slow down disease progression. Metabolic profiling revealed that Aβ triggered acute microglial inflammation, accompanied by metabolic reprogramming from oxidative phosphorylation to glycolysis through mTOR-HIF-1α pathway in cultured pure primary microglia. In 5 × FAD mice with chronic Aβ stimulation, microglia energy metabolism was impaired and subsequently, the immune response was diminished [130]. IFN-γ, which drives mTOR signaling and reverses mTOR-dependent loss-of-function effects, was used to boost metabolic pathways in vitro and in vivo [131, 132]. Baik et al. utilized IFN-γ to boost metabolic pathways and found that IFN-γ could decrease amyloid pathology and reverses memory deficits in 5 × FAD mice [130]. Metabolomics identified the mTOR-HIF-1α pathway in Aβ-triggered microglia inflammation, thus providing an ideal target for treatment. These studies further proved that modulation of microglial bioenergetic pathways by targeting the mTOR signaling pathway might be one of the strategies to treat AD.

Single-omics research is crucial; however, the integrated analysis of multi-omic data can better reveal the overall changes in disease pathogenesis, thus providing more valuable data for the development of therapeutic targets in human diseases. Two independent genomic studies first identified the *TREM2* variant R47H as a risk factor of LOAD in 2013 [6, 7]. Previous studies showed that *TREM2* was almost exclusively expressed in microglia in the brain, and mediated microglial phagocytosis and the response to inflammatory stimuli. However, the exact mechanism by which *TREM2* gene variants link microglia with the risk of AD was unknown. Recently, transcriptomic analysis by scRNA-seq in *Trem2*^+*/*+^ versus *Trem2*^*−/−*^ AD mice demonstrated that the development of DAM from the intermediate state to the final state required the activation of TREM2 [21]. snRNA-seq analysis of human *TREM2*-R47H and *TREM2*-R62H carriers with AD showed reduced transcriptional microglial activation compared with that in non-carriers [31]. These findings indicated that TREM2 plays an important role in the transition of microglia from the intermediate to the DAM state and emphasized a potential therapeutic strategy targeting TREM2-mediated microglia state transition. AL002c, a TREM2 agonistic monoclonal antibody, has been applied recently in a mouse model of AD [133]. scRNA-seq was performed to characterize the impact of AL002c on the microglial state in vivo. AL002c injection expanded subsets that were characterized by upregulated expression of genes associated with metabolic activation and proliferation, indicating that AL002c promoted microglia transition from a homeostatic state to DAM and proliferating microglia. Prolonged systemic administration of AL002c reduced filamentous plaques and neurite dystrophy, impacted behavior, and enhanced the numbers of neuroprotective microglia [133]. Moreover, a first-in-human phase I clinical trial has been conducted, which demonstrated the safety of a variant of AL002c (https://clinicaltrials.gov/ct2/show/NCT03635047).

A proteomic study found that many proteins were highly expressed in microglial cells in human AD brains. However, whether these abnormally expressed proteins are involved in the pathophysiology of AD remains a mystery. For example, RIPK1 was one of the highly expressed proteins in microglial cells in human AD brains [134]. RNA-seq of microglia isolated from AD mouse models (APP/PS1 mice and APP/PS1 RIPK1^D138N^ mice) demonstrated that RIPK1 mediated the transcriptional upregulation of *Cst7*, which encodes an endosomal/lysosomal cathepsin inhibitor, thus RIPK1 in turn impaired the microglial phagocytic capacity. Inhibition of RIPK1, using both pharmacological and genetic means, improved the behavioral deficits, reduced the neuroinflammation, and decreased the cerebral amyloid load by enhancing the microglial degradation of Aβ [134]. Thus, the transcriptomic approach provides a strong rationale for blocking RIPK1 as a novel therapeutic strategy for AD.

The above evidence demonstrated that integrated analysis of multi-omic data could mutually reinforce each other, provide solid and abundant evidence, and will eventually lead to further disease modifying therapies for AD.

### Targeting regulatory factors to induce specific microglia subsets

Signatures of microglia subsets in both AD mouse models and patients with AD have been identified; however, the regulatory or driving factors for a specific subset of microglia remain unclear. Grubman et al. utilized Single-Cell Regulatory Network Inference and Clustering (SCENIC), a computational method for simultaneous gene regulatory network reconstruction and cell-state identification from scRNA-seq data [135], to identify transcription factors inducing the signature formation of amyloid plaque-containing microglia (XO4 + microglia) [136]. SCENIC identified Hif1α as having the highest transcription factor-influence that contributed to the gene expression signature in XO4 + microglia. Furthermore, an in vitro assay validated the predicted regulatory function of HIF1α. Knockdown *Hif1a* using a short hairpin RNA (shRNA) in BV2 cells impaired the induction of the XO4 + gene signature by fibrillar Aβ treatment. Meanwhile, *Hif1a* overexpression promoted synaptosome phagocytosis of microglia in vitro. Moreover, Ingenuity Pathway Analysis (IPA) was performed to predict upstream small molecules to regulate Hif1α and its targeted genes: in human ESC-derived microglia-like cell lines, these predicted molecules (BMP9, MyD88, and mTOR) upregulated *HIF1A* mRNA, which in turn induced the network of genes associated with XO4 + microglia [136].

Recent scRNA-seq/snRNA-seq analyses revealed various subpopulations of microglia with differentially expressed gene sets. The function and impact of these microglial sub-populations in the CNS remain to be defined. Bioinformatic analysis is a powerful method to predict potential regulators of specific microglial subsets; however, more precise studies are required to validate these predictors by analyzing the expression levels of specific beneficial microglia subset signatures. These therapeutic strategies to control microglia fate towards a beneficial phenotype are worthy of exploration.

### Targeting the brain microenvironment to restore microglia function

The brain microenvironment exerts regulatory functions in the phenotype conversion of microglia. For instance, in a co-culture of organotypic brain slices of 20-month old APP/PS1 mice with young, neonatal wild-type (WT) mice, old microglia cells derived from 20-month-old APP/PS1 mice moved towards to the amyloid plaques and cleared the plaque halo. The capacity for proliferation and amyloid plaque phagocytosis of microglia could also be enhanced using conditioned media of young microglia or the addition of granulocyte–macrophage colony-stimulating factor [137]. This evidence indicated that microenvironment-driven restoration of microglia function could be a viable therapeutic strategy for AD. Previously, the microenvironment was largely unknown. However, spatial transcriptomics and in situ sequencing have provided insights linking cellular gene expression alterations to the Aβ load in AD [138]. Chen et al. investigated the transcriptional changes occurring around amyloid plaques using spatial transcriptomics in an AD mouse model. They found a gene co-expression network enriched for genes involved in myelination, which were mainly expressed by oligodendrocytes, was activated in the early stage with mild amyloid stress, but became depleted with high amyloid accumulation in the later stage. In contrast, a multicellular gene co-expression network involving inflammation, the complement system, oxidative stress, and lysosomes was notable in the later stage of the disease [138]. This spatial transcriptomics analysis untangled the molecular changes and cellular interactions in the vicinity of amyloid plaques of AD, uncovering the microenvironment around the amyloid plaques. In future, comprehensively determining the expression profiles of the cells surrounding specific microglia subsets would provide potential therapeutic targets for microenvironment modulation and microglia state regulation, thereby delaying or even halting disease progression.

## Biomarker development based on microglial “omics” analysis in AD

### Body fluid microglial biomarkers

Both genomic and transcriptome studies revealed that TREM2 plays a crucial role in AD pathogenesis. TREM2 undergoes proteolytic processing, releasing its ectodomain into the extracellular space as a soluble variant (sTREM2) via shedding by ADAM protease [139, 140], which can be detected in human plasma and CSF [141–143]. TREM2 is a key protein involved in the activation of microglia and AD mouse models have consistently found increased TREM2 expression during aging and disease progression [6, 144]; therefore, the question arises as to whether CSF sTREM2 would be an attractive candidate biomarker to track the disease. Below, we present a brief summary of findings that addressed the above question. Studies showed that the level of sTREM2 was significantly higher in the subjective cognitive decline group [145], the mild cognitive impairment (MCI) group [145] and the AD group [142, 143, 145–147] compared with that in healthy controls, and its level in AD was higher than that in MCI [145]. Notably, several years prior to the onset of symptoms, the level of CSF sTREM2 of dominantly inherited AD had already increased, and the increase was maintained after disease onset [148]. Most studies found that the CSF sTREM2 level correlated highly with markers of neurodegeneration, such as the CSF levels of total tau, phosphorylated-tau (p-Tau), and fibrillar tau pathology [142, 143, 145, 146]. However, whether CSF sTREM2 is related to Aβ pathology is still inconclusive [148–150]. The pathological roles of sTREM2 in AD have been studied by direct stereotaxic injection of recombinant sTREM2 protein or by adeno-associated virus-mediated expression of sTREM2 in the brain of 5 × FAD mice. The results showed that sTREM2 reduced amyloid plaques, and improved spatial memory impairment and long-term potentiation in the AD mouse model [151]. Mechanistic studies further found that sTREM2 promoted the proliferation and activation of microglia, increased the migration of microglia around amyloid plaques, and enhanced microglia phagocytosis of Aβ [151, 152]. This evidence suggested that CSF sTREM2 might be a biomarker for microglia activation.

Quantitative mass spectrometry analysis identified a set of microglial proteins that were differentially abundant in patients with AD, and available evidence supported the view that these differentially abundant microglial proteins could be detected in the CSF. Johnson et al. performed a quantitative mass spectrometry analysis of dorsolateral prefrontal cortex tissue of 453 control, asymptomatic AD, and AD brains. Co-expression network analysis constructed a 13 ‘module’-containing protein co-expression network. Module 4 (M4), which was enriched in microglial and astrocyte proteins demonstrated maximal alterations in patients with AD and correlated most significantly with AD pathology and cognitive impairment [112]. In the same study, CSF samples from two independent cohorts (one cohort of 297 subjects consisting of controls and patients with ADs, and a second cohort of 96 subjects consisting of control, AsymAD, and AD) were also analyzed. The proteins in M4 were also increased in the CSF of AD and asymptomatic subjects [112].

Besides, Kim et al. performed liquid chromatography coupled to tandem mass spectrometry (LC–MS) analysis in the hippocampus and identified proteins that were differentially abundant between WT and 5 × FAD mice, in which vitamin K-dependent protein S (PROS1) was increased in the hippocampus of 5 × FAD mice at 10 months of age [153]. PROS1 is mainly produced by microglia in the brain, and is likely to be closely associated with amyloid pathology. An in vitro study found that treatment with Aβ monomers induced primary microglia secretion and the secreted PROS1 might further promote the phagocytic activity of microglia [153]. Importantly, the level of PROS1 in serum was related to disease progression. In 5 × FAD mice, as the disease progressed, the level of PROS1 in serum increased. In patients with AD, the level of PROS1 in serum was also significantly higher than that in the healthy control and MCI groups. Combined with amyloid imaging using Pittsburgh compound ([^11^C]PIB) positron emission tomography (PIB-PET), it was further confirmed that the level of PROS1 in serum could reflect the deposition of Aβ in human brains [153]. Therefore, these findings suggest that serum PROS1 is expected to be a novel microglia-derived biomarker to monitor disease progression.

Through proteomic analysis of AD mouse models and post-mortem brain tissues of patients with AD, some microglia-specific and highly expressed proteins have been identified, such as Msn, Cotl1, and Siglec-8. These proteomic profiles of microglia provide a potential source of biomarkers; however, whether these highly expressed proteins can be detected in CSF and peripheral blood, and whether they can reflect the same trend of changes in brain tissue samples require further study.

### Microglial extracellular vesicles (EVs) might serve as source of biomarkers

EVs, existing in almost all body fluids, including CSF and blood, mediate cell-to-cell communication. Microglia-derived EVs, consist of proteins, RNAs, and lipids, are a novel source of biomarkers to monitor pathology progression. In the CNS, EVs have been suggested as potential carriers that propagate misfolded proteins associated with neurodegenerative disorders, such as tau and Aβ in AD and α-synuclein in PD [154]. For example, a study by Clayton et al. showed that in a humanized APP mouse model, MGnD microglia hyper-secrete phosphorylated (p)-Tau-encapsulating EVs, thereby accelerating tau propagation [155]. Moreover, inhibiting microglia secretion of tau-containing EVs alleviated cognitive impairment and tau pathology in P301S tau transgenic mice [156]. Quantitative proteomic analysis of brain-derived EVs from an AD mouse model in the CAST/EiJ strain identified 3444 unique proteins [157]. Compared with WT-derived EVs, CAST.APP/PS1-derived EVs showed significant enrichment of integrin Itgax and Apoe, which are markers of microglia DAM/MGnD subsets, demonstrating that DAM/MGnD play critical roles in EVs secretion in the brains of AD mouse models [157]. A recent study used multi-omics, including proteomics, lipidomics, and NanoString nCounter technology, to analyze microglial CD11b^+^ small EVs from parietal cortex tissues of four late-stage AD (Braak V–VI) and three age-matched normal/low pathology cases [124]. Compared with control cases, CD11b^+^ EVs from AD brains showed a reduction in the abundance of homeostatic microglia markers P2RY12 and TMEM119, and increased levels of DAM markers ferritin heavy chain-1 (FTH1) and TREM2. Levels of free cholesterol were also elevated in microglial EVs from the AD brains [124]. Thus, these findings indicated that microglial EVs from AD brain tissues revealed DAM signatures. EVs are regarded as novel diagnostic and prognostic biomarkers for many diseases. Currently, there is a lack of widely accepted specific markers of microglia-derived EVs in clinical application, and even more so for the specific subsets of microglia. However, future extraction of microglia-derived exosomes from CSF, combined with multiple omic analysis, has merit to identify novel EV-associated biomarkers.

### Potential imaging biomarkers for microglia

Translocator protein 18 kDa (TSPO) is mainly expressed in the mitochondrial outer membrane of microglia, and its expression is remarkably increased when microglia are activated. Positron emission tomography (PET) imaging of TSPO is a strategy to detect microglial activation in vivo. However, this method has its limitations. Firstly, TSPO is also expressed by other cell types, such as astrocytes and endothelial cells [158], indicating its non-specificity for activated microglia. Secondly, human TSPO polymorphisms affect the binding affinities of second-generation tracers [159]. Thirdly, and more importantly, the TSPO tracers are unable to differentiate between distinct microglia phenotypes [160]. For example, Fan et al. conducted a longitudinal study to evaluate the temporal profile of microglia activation at baseline and at 14 ± 4 months of follow-up in 30 subjects (8 MCI, 8 AD, and 14 healthy controls). Compared with that in the controls, microglia activation was increased in the MCI and AD groups, both at baseline and after follow-up. However, during follow-up, the TSPO PET signal intensity of microglia activation decreased compared with that at baseline in the MCI group, while it increased continuously in the AD group. One hypothesis was that activated microglia showed a protective phenotype in the early MCI stage; however, the protective activated microglia decreased with disease progression, thus the results showed that the longitudinal PET TSPO signal of MCI decreased compared with that at baseline, although it still showed an increased signal compared with that in the healthy controls. However, in the AD stage, the activated microglia had a pro-inflammatory impact; therefore, the PET TSPO signal increased continuously [160]. Thus, there might be one early protective peak and one later pro-inflammatory peak of microglial activation in the AD trajectory. Although the microglia phenotypes in MCI and AD are distinct, both of these different microglia phenotypes showed a significantly increased TSPO signal compared with that in the controls. Thus, TSPO tracers cannot be used to discriminate between different microglia phenotypes. Unfortunately, accurate biomarkers for in vivo microglia subsets with different phenotypes have not been discovered yet. Currently, the comprehensive characterization of microglia subsets in multiple brain regions has become possible by combining massively parallel single-cell analysis, single-molecule fluorescence in situ hybridization, advanced immunohistochemistry, and computational modelling [161]. Once molecular surface markers of specific temporal and spatial microglia subsets are identified, corresponding PET tracers will be developed. In this way, we could presage disease progression, and predict and evaluate the therapeutic effects of microglia-targeting drugs by observing the dynamic alterations of microglia subsets in vivo.

## Spatial transcriptomics, a promising tool to reveal intercellular communication

### Microglia communicate with other cell types in the AD brain

#### Microglia-astrocyte crosstalk

The influence of microglia and astrocytes on each other produces both beneficial and detrimental effects. Activated microglia induce the transformation of astrocytes into the A1 phenotype by releasing pro-inflammatory cytokines interleukin-1a (IL-1a), tumor necrosis factor-alpha (TNF-α), and complement component1q (C1q). A1 astrocytes, assumed to be a reactive neurotoxic form, lose their ability to provide trophic support, clear debris, and execute phagocytosis, finally leading to the death of neurons and oligodendrocytes [162]. Alternatively, microglia can assist astrocytes to clear internalized pathological aggregates. HiPSC-derived astrocytes and microglia were exposed to αSYN or Aβ fibrils. Co-cultures of astrocytes and microglia significantly reduced intracellular αSYN and Aβ deposits compared with monocultures of either cell type. Astrocytes secreted internalized protein aggregates and microglia attached to the astrocyte cell membrane and phagocytosed αSYN and Aβ deposits [163].

Different studies have reported that astrocytes can both facilitate and inhibit microglia phagocytosis of Aβ. McAlpine et al. found that upon recognition of Aβ deposits, microglia increased their expression of IL-3Rα, which is the specific receptor for IL-3. Astrocyte-derived IL-3 binds to the upregulated IL-3Rα in microglia, which elicits the immune response of microglia, manifested by enhanced motility, migration towards, and cluster around, Aβ deposits, and the clearance of Aβ aggregates [164]. However, Lian et al. reported that neuronal overproduction of Aβ activated NF-κB signaling in astrocytes, which in turn promoted the release of complement C3. C3, secreted from astrocytes, interacts with the microglial C3a receptor (C3aR) and compromises microglial Aβ phagocytosis, resulting in increased Aβ deposition and cognitive decline in AD mouse models [165]. Besides, a recent study demonstrated that astrocyte-derived APOE contributed to microglia-dependent synaptic phagocytosis in tau transgenic mice. In the brain, APOE is mainly produced by astrocytes. The tau transgenic mice were administrated with tamoxifen to decrease astrocytic APOE. Removal of astrocyte-specific APOE transformed astrocytes from an activated to a more homeostatic state. Meanwhile, snRNA-seq revealed that disease-associated gene signatures in microglia were decreased. In the tau transgenic mice, microglia engulfed synapses and reduced synapse density. However, removal of astrocytic APOE significantly attenuated synapse phagocytosis by microglia, and reduced tauopathy and tau-mediated neurodegeneration [166].

#### Microglia-cerebral blood vessels crosstalk

The neurovascular unit (NVU) comprises functionally interacting cells, including neurons, glial cells, and vascular cells, which are linked to each other to enable an effective blood–brain barrier (BBB). Microglia adjacent to the BBB constantly survey the integrity of the BBB and repair the damaged BBB via bidirectional communication with other NVU cells [167–169]. A recent study demonstrated that in the healthy adult brain, microglia can interact with the vasculature to regulate vascular structure and function [170, 171]. Bisht et al. discovered a class of microglia whose soma resided on the vasculature, called capillary-associated microglia (CAM). The P2RY12 receptor is expressed on microglia and the ATP permeable integral membrane protein PANX1 is expressed on capillaries. Purines released from PANX1 on capillaries attract and maintain microglia at the capillary wall, where microglia regulate cerebrovascular perfusion and reactivity through PANX1-P2RY12 coupling [171]. How microglia affect vascular function in neurodegenerative diseases raises exciting questions that will be addressed by future studies. Breakdown of the BBB and increased permeability have been observed in patients with AD and the disruption of BBB correlates with disease progression [172]. Compromised vascular integrity leads to the influx of blood proteins into the brain, and these proteins might be neurotoxic and aggravate neurodegeneration [173]. Fibrinogen, a blood coagulation protein that leaks through the BBB, is deposited in proximity to the eliminated dendritic spine in the AD brain. Fibrinogen binds to CD11b on microglia to induce microglia activation, which in turn induces spine elimination and promotes cognitive deficits. Genetic elimination of the fibrinogen binding motif of CD11b reduced microglia activation, synaptic deficits, and cognitive impairment in AD transgenic mice [174]. Overall, microglial dysfunction might lead to BBB damage through the diminished ability to repair the BBB or by directly affecting vascular structure and function. In turn, the damaged BBB could cause substances to leak into the CNS to promote microglial activation and accelerate neurodegeneration.

#### Microglia-neuron crosstalk

Precise regulation of synapse formation and elimination is critical for learning and memory, and microglia-neuron interactions are involved in this process. Microglia can prune synapses via the complement pathway. C3 and C1q are extensively expressed and localize to excessive synapses, sending the “eat me” signal. Microglia recognize the "eat me" signal, and microglial complement receptor CR3 binds to neuronal C3, promoting synapse engulfment by microglia [73, 175, 176]. Abnormal upregulation of adenosine A2A receptor (A2AR) has been observed in AD and ageing populations. Neuronal A2AR upregulation led to a hippocampal upregulation of C1q complement in a tauopathy mouse model, which promoted synaptic loss and memory deficits [176]. In contrast, inhibition of C1q, C3, or CR3 reduced the number of phagocytic microglia, as well as rescued synaptic loss and dysfunction [73]. Meanwhile, some mechanisms protect synapses from excess pruning by microglia. CD47 localize to synapses and send the “don’t eat me” signal. SIRPα is CD47 receptor expressed on microglia. Microglia recognize the "don’t eat me" signal by CD47-SIRPα signaling. Mice with *Cd47* knockout demonstrated increased microglial engulfment of synapses, and a sustained reduction in synapse numbers [177]. In addition, microglia-specific deletion of *Sirpa* also resulted in increased synaptic loss mediated by microglia engulfment and enhanced cognitive impairment in AD mouse models [178]. Importantly, SIRPα levels were remarkably decreased in the cortexes of patients with AD and AD mice brains compared with that in age-matched controls and microglial SIRPα expression decreased together with disease progression in AD mice [178]. These results suggest that the balance of “eat me” and “don't eat me” signals associated with synaptic pruning by microglia is disrupted in AD, resulting in excessive synapse loss and neuronal dysfunction.

### Spatial transcriptomics reveal intercellular communication in the microenvironment

Cell communication is a fundamental process of multicellular organisms. To respond to the external environment properly, cells have developed complex mechanisms of communication such that they can receive and transfer the message, and then generate changes within the cell in response to the message. Therefore, understanding cell-to-cell communication under physiological and pathological conditions is crucial to determine disease pathogenesis and identify therapeutic strategies. The availability of single-cell transcriptomics offers an exciting opportunity to gain an insight into changes in individual cells. However, false positives are a possibility when using scRNA-seq data to analyze cell-to-cell interactions [179, 180]. For example, by identifying membrane bound ligand and receptor expression in different cell subsets, it is possible to discover two cell subsets that interact with each other; however, because of the lack of spatial information in scRNA-seq data, these two cell subsets might actually be spatially distant and cannot interact [179, 180]. The relative stability of cellular locations mean that spatial transcriptomics can reveal cell–cell communications with fewer false positives than similar analysis using scRNA-seq data. Using spatial transcriptome and analysis tools, interactions between cells that only have ligand-receptor co-expression, but without the physical possibility for communication between, them will be filtered out [180].

Currently, spatial transcriptomic approaches alone cannot provide deep transcriptomic information at the single-cell level; however, they can identify distinct gene sets that are enriched in the analyzed niches. In these niches, different cell populations communicate with each other through ligand–receptor interactions or secreted substances. When spatial transcriptomes are combined with scRNA-seq, we can localize transcriptionally characterized single cells within the niche [181]. This is expected to facilitate further progress, for example: (1) Capturing the spatial distribution of cell subpopulations in the brain and revealing local networks of intercellular communication among neurons, microglia, and oligodendrocytes in development, homeostasis, and disease; (2) monitoring dynamic changes in cellular subpopulation composition in the microenvironment during disease progression; (3) combined with in situ sequencing, untangling the molecular changes and cellular interactions in the vicinity of amyloid plaques or other neuropathological markers of AD [138]. For example, spatial transcriptomics and in situ sequencing demonstrated that in the early stage with a mild Aβ load, oligodendrocytes might be the first cells to show altered gene expression profiles that are enriched for genes involved in myelination. However, in the advanced stages of the disease, a multicellular gene co-expression network involving inflammation, the complement system, oxidative stress, and lysosomes was detected [138]. Although the spatial transcriptome technique is still in its infancy and has some limitations, it holds promise as a means by which we can determine cell–cell interactions in the microenvironment.

## Conclusions

While the pathological roles of microglia in AD have been studied for decades, our understanding of the heterogeneity and dynamics of microglial responses during AD progression remains poor. The advent of high throughput omic data analyses, especially scRNA-seq/snRNA-seq techniques, facilitates the identification of special AD-associated microglial subsets. Currently, most of the microglia subsets have been defined by transcriptional profiling, and thus determination of the functional roles of these microglia subsets should be basis for the development of therapeutic strategies. Clarification of the dynamic changes in microglia subsets during disease progression is an essential prerequisite for disease monitoring and treatment. In addition, analysis of spatial information of specific microglia subsets in the brain will facilitate the interpretation of functions and the understanding of the subset microenvironment. These issues are research priorities that should be addressed before future large-scale clinical applications. Although research into microglia subsets is still in its infancy, emerging microglia subsets could lead to the development of novel therapeutics and biomarkers in AD. Besides microglia subset identification, microglial omic studies are helpful to discover dysregulated pathways and key molecules that are critical for AD pathogenesis, and will promote the development of new therapeutic strategies. Meanwhile, these omic studies provide a source to develop biomarkers to monitor disease progression (Fig. 1).

With this in mind, the most important goal for the next decade of research into microglia in AD should include: (i) understanding the functional roles of novel identified microglia subsets; (ii) using bioinformatic analyses to identify regulatory factors of specific microglia subsets; (iii) using human iPSC differentiation into microglia in combination with molecular genetic techniques (e.g., CRISPR) to study the pathogenic roles of AD-associated variants; (iv) validation of newly discovered microglia-specific highly expressed proteins in CSF and blood; (v) using spatial transcriptomics to reveal microglial communication with other cell types; and (vi) last but not least, the generation of chimeric mice using xenotransplantation to recapitulate human microglial biology in vivo [182].

## Abbreviations

AD: Alzheimer’s disease
Aβ: Amyloid β
GWAS: Genome wide association studies
TREM2: Triggering receptor expressed on myeloid cells 2
ApoE: Apolipoprotein E
CNS: Central nervous system
NLRP3: NACHT-, LRR- and pyrin (PYD)-domain-containing protein 3
ASC: Apoptosis-associated speck-like protein containing a caspase activation and recruitment domain (CARD)
LPS: Lipopolysaccharide
IL: Interleukin
scRNA-seq: Single-cell RNA sequencing
snRNA-seq: Single-nuclei RNA sequencing
DAM: Disease-associated microglia
WAM: White matter-associated microglia
LOAD: Late-onset AD
ITAM: Immunoreceptor tyrosine-based activation motif
DAP12: DNAX-activating protein of 12 kDa
Syk: Spleen tyrosine kinase
MS4A: Membrane-spanning 4-domains subfamily A
CSF: Cerebrospinal fluid
sTREM2: Soluble TREM2
ABCA7: ATP-binding cassette transporter A7
CR1: Complement receptor 1
SNPs: Single-nucleotide polymorphisms
BIN1: Bridging Integrator 1
HiPSCs: Human induced pluripotent stem cells
PLCG2: Phospholipase C gamma 2
PIP2: 1-Phosphatidyl-1D-myo-inositol 4,5-bisphosphate
IP3: Myo-inositol 1,4,5-trisphosphate
DAG: Diacyl-glycerol
DMRs: Differentially methylated regions
MAMSTR: MEF2-activating motif and SAP domain-containing transcriptional regulator
MEF2C: Myocyte enhancer factor-2C
FACS: Fluorescence-activated cell sorting
MACS: Magnetic-activated cell sorting
ALS: Amyotrophic lateral sclerosis
ARM: Amyloid-responsive microglia
WT: Wild-type
Msn: Moesin
AsymAD: Asymptomatic AD
PD: Parkinson’s disease
SCENIC: Single-cell regulatory network inference and clustering
IPA: Ingenuity pathway analysis
MCI: Mild cognitive impairment
p-Tau: Phosphorylated-Tau
LC–MS: Liquid chromatography coupled to tandem mass spectrometry
PROS1: Vitamin K-dependent protein S
PIB-PET: Pittsburgh compound ([11C]PIB) positron emission tomography
EVs: Extracellular vesicles
FTH1: Ferritin heavy chain-1
TSPO: Translocator protein 18 kDa
